# Oral and dental health care practices in pregnant women in Australia: a postnatal survey

**DOI:** 10.1186/1471-2393-8-13

**Published:** 2008-04-21

**Authors:** Natalie J Thomas, Philippa F Middleton, Caroline A Crowther

**Affiliations:** 1 Discipline of Obstetrics and Gynaecology, The University of Adelaide, SA, Australia

## Abstract

**Background:**

The aims of this study were to assess women's knowledge and experiences of dental health in pregnancy and to examine the self-care practices of pregnant women in relation to their oral health.

**Methods:**

Women in the postnatal ward at the Women's and Children's Hospital, Adelaide, completed a questionnaire to assess their knowledge, attitudes and practices to periodontal health. Pregnancy outcomes were collected from their medical records. Results were analysed by chi-square tests, using SAS.

**Results:**

Of the 445 women enrolled in the survey, 388 (87 per cent) completed the questionnaire. Most women demonstrated reasonable knowledge about dental health. There was a significant association between dental knowledge and practices with both education and socio-economic status. Women with less education and lower socio-economic status were more likely to be at higher risk of poor periodontal health compared with women with greater levels of education and higher socioeconomic status.

**Conclusion:**

Most women were knowledgeable about oral and dental health. Lack of knowledge about oral and dental health was strongly linked to women with lower education achievements and lower socioeconomic backgrounds. Whether more intensive dental health education in pregnancy can lead to improved oral health and ultimately improved pregnancy outcomes requires further study.

## Background

The most important objective of dental health care in pregnancy is to establish a healthy environment through adequate plaque control by brushing, flossing and professional prophylaxis including scaling, root planing and polishing [1]. Dental treatment can be safely provided at any time during pregnancy [2] allowing pregnant women to achieve an optimal level of dental health throughout their pregnancy.

Periodontal disease may present as gingivitis or periodontitis. Gingivitis is an inflammation of the soft tissues surrounding a tooth or gingiva not causing loss of periodontal attachment, whereas periodontitis causes inflammation and destruction of supporting tissues around the teeth [2]. Periodontal disease has the potential to affect pregnancy outcomes. A systematic review of 25 studies (13 case-control, 9 cohort and 3 controlled trials) has demonstrated that periodontal disease may be associated with adverse pregnancy outcomes in humans [3]. Although some observational studies have indicated a significant association of periodontal disease with adverse pregnancy outcomes [4,5], others have not [6,7].

Oral tissues are known to be affected by pregnancy with the most frequent and greatest changes occurring in the gingival tissue [8]. Pregnant women may be more susceptible to periodontal disease since higher concentrations of oestrogen and progesterone can induce hyperaemia, oedema and bleeding in periodontal tissues [9], increasing the risk of bacterial infections. The incidence of periodontal disease has been positively correlated with lower educational achievement and lower socio-economic status [9-11].

Periodontal disease is both preventable and treatable. Controlling plaque by brushing, flossing and professional prophylaxis, including scaling and root planing, all help to achieve good dental health in pregnancy [1]. There is, however, minimal information available on women's understanding of dental hygiene and whether pregnant women comply with current oral health strategies.

The aims of this survey were to assess women's knowledge and experiences about dental hygiene in pregnancy in Australia and assess the self-care practices of pregnant women in relation to their oral health. We hypothesised socio-economic status and educational qualifications would influence a woman's knowledge and choices about oral health in pregnancy.

## Methods

All women who gave birth to a live born infant at the Women's and Children's Hospital, Adelaide, over a five month period were eligible for the study. Women who gave birth to an infant with a major congenital abnormality, had a perinatal death or who needed an interpreter were ineligible. The study was approved by the Hospital Research and Ethics Committee.

A questionnaire was developed to assess women's dental health knowledge, oral health experiences and preferences over the preceding twelve months and provide information on their educational status. Fifty questions were selected from three validated questionnaires; the National Dental Telephone Interview Survey (NDTIS) [12], Oral Health Impact Profile (OHIP) [13] and The World Health Organization's Comparing Oral Health Care systems; a second international collaborative study (ICS II) [14]. Additional questions included information in relation to awareness of periodontal disease and plaque, use of a family dentist, advice about dental health requirements during pregnancy, history of bleeding gums and what, if any, actions were sought to treat perceived gingival problems. Relevant clinical information, such as pregnancy care, parity, ethnicity, age and residential location, was sourced from the medical records of the woman and her infant.

Eligible women were approached on the postnatal ward and given a study information pamphlet. Women who gave informed written consent were asked to complete the questionnaire and place it in the reply paid envelope. Women who failed to return the questionnaire within three weeks were posted out a reminder letter and another questionnaire. Women who did not return the questionnaire were telephoned as a reminder.

Analysis was performed with SAS [15]. Descriptive statistics were reported as well as cross-tabulations by age, parity, education and socio-economic status. Inference on the cross-tabulations was performed, using chi-square tests to test for general association and Mantel-Haenszel chi-square tests to test for linear association where appropriate.

In order to show a significant difference between women with a tertiary education compared with women without a tertiary education who brushed their teeth at least daily, a sample size of 374 women was needed. This was calculated estimating a difference of 9% between groups based on 95 per cent of mothers who had a tertiary education and 86 per cent of mothers who did not have a tertiary education (p < 0.05; 80% power) would brush their teeth at least daily. The sample size was adjusted upward to allow for a 14 per cent non-completion rate.

During the enrolment period a total of 505 eligible women were approached; 445 (88%) consented and 388 (87%) returned their questionnaire. Although small numbers of responses were missing from some questions, a denominator of 388 was used to calculate results (except in the cross-tabulation in Table 1).

**Table 1:** Dental health of women completing the postnatal survey

***Perceived dental problems in previous 12 months***				**n (%)**	
*Broken or chipped natural tooth*				73 (18.8)	
*Gums that hurt or bleed*				231 (59.5)	
*Sores on tongue or inside mouth*				58 (14.9)	
*Bad taste in mouth*				103 (26.5)	
*Persistent bad breath*				50 (12.9)	

***Current dental health problems caused***	***often n (%)***	***occasionally n (%)***	***rarely n (%)***	***never n (%)***	***don't know n (%)***

*Pain/discomfort*	47 (12.1)	98 (25.3)	107 (27.6)	120 (30.9)	7 (1.8)
*Limitation*	15 (3.9)	34 (8.8)	40 (10.3)	292 (75.2)	0
*Uncomfortable to eat*	39 (10.1)	66 (17.0)	67 (17.3)	210 (54.1)	5 (1.3)
*Unsatisfactory diet*	11 (2.8)	19 (4.9)	31 (8.0)	314 (80.9)	10 (2.6)
*Embarrassment*	30 (7.7)	38 (9.8)	31 (8.0)	282 (72.7)	6 (1.6)
*Less satisfying life*	20 (5.2)	23 (5.9)	27 (7.0)	312 (80.4)	4 (1.0)

## Results

### Baseline demographic characteristics

Women who returned the survey were more likely to be older (p < 0.05) than non-respondents. Just over half of the women enrolled in the study were over the age of 30 (n = 219, 56%). The majority of women who completed the questionnaire were caucasian (344, 89%). About half were in their first pregnancy (185, 48%), had some form of tertiary education (199, 51%) and lived in a low to mid socio-economic index area (216, 56%) (Table 2).

**Table 2:** Demographics of women who completed the survey

	**Completed survey (388 women) n (%)**
***Age (years)***	
*< 20*	10 (3)
*20 – 29*	159 (41)
*30+*	219 (56)
***Parity***	
*0*	185 (48)
*1–3*	187 (48)
≥ *4*	16 (4)
***Race***	
*Caucasian*	344 (89)
*Asian*	19 (5)
*Aboriginal/TSI*	11 (3)
*Other*	14 (4)
***Education***	
*Not completed secondary school*	76 (20)
*Completed secondary school*	113 (29)
*TAFE/diploma*	91 (23)
*University*	108 (28)
***Socio-economic status***	
*Low SEI*	109 (28)
*Low-mid SEI*	107 (28)
*Mid-high SEI*	67 (17)
*High SEI*	105 (27)

TSI = Torres Strait Islanders; SEI = socio-economic index

### Knowledge of dental practices

Most women had a good understanding of good oral hygiene, with 382 (99%) women agreeing brushing their teeth would help prevent gum disease. Likewise, most women understood using dental floss (325, 84%) would help prevent gum problems. The majority of the women surveyed knew that fluoride, whether in toothpaste (350, 90%) or water (310, 80%), helped to prevent tooth decay.

Women with a university education were more likely than other women to strongly agree that the use of dental floss would help prevent gum and tooth problems (p < 0.0001). Women with lower educational levels knew less about the beneficial effects of fluoride toothpaste (p < 0.01), fluoridated water (p < 0.001) and the ability of fluoride to prevent tooth decay without harm (p < 0.01). Women with a university education had better knowledge of periodontal disease (p < 0.001) and dental plaque (p < 0.03). Women in the high socio-economic index (SEI) were more likely than other women to strongly agree that the use of dental floss would aid in the prevention of gum problems (p < 0.02).

Women above thirty years of age were more likely to strongly agree that the use of fluoridated water (p < 0.001) helps prevent tooth decay, to know about periodontal disease (p < 0.04) and to use dental floss more often then younger women (p < 0.03). Higher parity was associated with greater knowledge of fluoridated water preventing tooth decay (p < 0.02) and fluoride preventing tooth decay (p < 0.02).

### Knowledge of dental disease and gingival health

Most women had some knowledge of dental disease and gingival health with the majority of women surveyed agreeing sweet foods could cause tooth decay (326, 84%). Likewise, they were aware that dental problems can be serious (387, 100%) and can cause other health problems (355, 92%). Although the majority of women knew about dental plaque (366, 94%), 317 (82%) of the women surveyed did not know about periodontal disease.

### Current dental practices

Of the women surveyed, 351 (91%) stated they brushed their teeth one or more times a day with just over half (222, 57%) indicating they used dental floss weekly or more. Just over half (221, 55%) of women said that they used mouthwash more than once a month. Tertiary educated women were also more likely to brush their teeth once or more a day and to use dental floss (p < 0.001) (Table 3).

**Table 3:** Dental hygiene practices of women by education achieved

***EDUCATION***	***never n (%)***	***monthly n (%)***	***weekly n (%)***	***daily+ n (%)***	***p-value***
***How often teeth were brushed***					
- not completed secondary school	1 (1)	1 (1)	14 (18)	60 (79)	
- completed secondary school	0	2 (2)	10 (9)	101 (89)	
- TAFE/diploma	0	0	6 (7)	85 (93)	
- University	0	0	3 (3)	105 (97)	<0.0001
					
***How often dental floss was used***					
- secondary school not completed	29 (38)	15 (20)	25 (33)	7 (9)	
- secondary school completed	33 (29)	18 (16)	44 (39)	18 (16)	
- TAFE/diploma	25 (27)	10 (11)	47 (52)	9 (10)	
- University	23 (21)	13 (12)	57 (53)	15 (14)	0.005
					
***How often mouthwash was used***					
- secondary school not completed	34 (45)	14 (18)	14 (18)	14 (18)	
- secondary school completed	56 (50)	14 (12)	21 (19)	22 (20)	
- TAFE/diploma	35 (39)	10 (11)	24 (26)	22 (24)	
- University	49 (46)	21 (20)	9 (9)	27 (26)	0.074

+ more than daily

Almost two thirds (252, 65%) of the women surveyed found problems with their teeth, dentures or gums had caused some pain and/or discomfort during the previous twelve months. For the majority of women (292, 75%) these problems did not cause any limitations of their usual activities (Table 1). Just under half of the women (172, 44%) indicated it was uncomfortable to eat some foods on one or more occasions but this did not cause their diet to become unsatisfactory (314, 81%). The majority thought any problems with their teeth, dentures or gums during the preceding twelve months had not caused them any embarrassment (282, 73%) or made their life less satisfying (312, 80%) (Table 1).

The majority of women found their general health to be above average (334, 86%) with 158 (41%) believing it to be very good. However, more women rated their dental health as poor (40, 10%) compared with their general health (1, 0.3%). Women who had completed secondary school were less likely to have bleeding gums during the preceding 12 months (p < 0.004). High SEI women were more likely to agree fluoride toothpaste would prevent tooth decay (p < 0.04) and dental problems could be serious (p < 0.001). They brushed their teeth more than lower SEI women (p < 0.001) and were more likely to use dental floss than lower SEI women (p < 0.02) (Table 4).

**Table 4:** Dental hygiene practices of women by socio-economic status

***SOCIO-ECONOMIC INDEX***	***never n (%)***	***monthly n (%)***	***weekly n (%)***	***daily+ n (%)***	***p-value***
***How often teeth were brushed***					
- low SEI	1 (1)	2 (2)	17 (16)	89 (82)	
- low-mid SEI	0	0	7 (7)	100 (93)	
- mid-high SEI	0	0	5 (7)	62 (93)	
- High SEI	0	1 (1)	4 (4)	100 (96)	<0.001
					
***How often dental floss was used***					
- low SEI	41 (38)	16 (15)	37 (34)	15 (14)	
- low-mid SEI	30 (28)	14 (13)	50 (47)	13 (12)	
- mid-high SEI	17 (25)	16 (24)	27 (40)	7 (10)	
- high SEI	22 (21)	10 (10)	59 (56)	14 (13)	0.019
					
***How often mouthwash was used***					
- low SEI	46 (42)	15 (14)	17 (16)	31 (28)	
- low-mid SEI	51 (48)	17 (16)	18 (17)	21 (20)	
- mid-high SEI	34 (51)	11 (16)	12 (18)	10 (15)	
- high SEI	43 (42)	16 (16)	21 (20)	23 (22)	0.238

+ more than daily

### Dental attendance and dental problems in the past 12 months

During the previous twelve months 193 (50%) women surveyed had attended the dentist with 91 (24%) of these women attending only once in this period. One third of women, 129 (33%), had at least one scaling and cleaning of their teeth during these dental visits.

The majority of women, (231, 60%) stated they had gums which hurt and/or bled at some stage during the previous twelve months and 103 (27%) had a bad taste in their mouth. Only 50 (13%) had persistent bad breath and 58 (15%) women had sores on their tongue and/or inside their mouth. Only 73 (19%) women had a broken or chipped natural tooth during the previous 12 months (Table 3).

How women addressed these reported dental problems varied. Some women intensified their oral hygiene (173, 45%), while others increased the use of oral rinse products (93, 24%) or visited the dentist (47, 12%). However 86 (22%) women chose to take no action.

Although 250 (64%) women were advised of dental health requirements during pregnancy, only 116 (30%) women attended the dentist once or more whilst pregnant. Most women were informed about dental health in pregnancy through conversations with their dentist (165, 43%), through reading material (116, 30%) and talking with their midwife or doctor (54, 14%).

Of the women surveyed, 239 (62%) stated they required a dental check-up with 174 (45%) needing scaling and cleaning of their teeth. Only 39 (10%) women stated they thought they needed gum treatment. At least one filling was needed by 124 (32%) women with 43 (11%) women needing teeth extracted.

## Discussion

In this survey of recently pregnant mothers, most women were knowledgeable about dental health but only a small percentage knew about periodontal disease.

Over half of the women surveyed revealed they did not attend the dentist during the previous twelve months, and only 30% attended during their most recent pregnancy. The majority indicated they required a dental check-up with just under half believing they needed scaling and cleaning of their teeth. The results from this survey are consistent with studies conducted in the United States of America where more than 50% of pregnant women did not receive dental care during their most recent pregnancy [11,16]. This raises serious concerns about dental care-seeking behaviours as most adults would be due for their routine dental visits during any nine-month period, and pregnant women may need extra periodontal care [1]. Over half the women surveyed (65%) recalled being informed of the dental health requirements in pregnancy yet they did not attend the dentist.

Cost of dental care may have an impact on the dental seeking behaviours of pregnant women in Australia. In Australia women must have insurance or be prepared to pay to cover private dental treatment or be placed on a waiting list to seek free treatment in the public system. This could be an independent risk factor whether pregnant women seek dental advice and treatment or not [17].

Many women surveyed reported signs suggestive of periodontal disease during the previous twelve months by the occurrence of bleeding, sore gums and a persistent bad taste in their mouth. Our findings confirm bleeding and sore gums are common among pregnant women [9,14]. Although women indicated they had suffered discomfort from sore and bleeding gums and had bad taste in their mouth only a small proportion chose to attend the dentist.

This survey highlights important gaps in dental knowledge and practices in women, particularly those with lower educational achievements and lower socio-economic status. Better knowledge of dental hygiene and practices were found in women who had some form of tertiary education and from a higher socio-economic status. Similar results were found in the recent systematic review of 25 studies [3]. Failure to attend a dentist on a regular basis and lack of understanding about the importance of maintaining oral hygiene may be because some women simply cannot afford to maintain an adequate level of dental hygiene or regular dental visits. Educating and motivating women to maintain good oral hygiene and providing affordable dental health care is fundamental in reducing dental disease. Improving dental education may need to become a priority in antenatal care to educate women at risk of the importance of maintaining oral health [18].

Although this study has the limitations of relying on self-reported data and therefore is subject to biases inherent to this method, such as misclassification of the question being asked, most women completed the study within one to three days of birth therefore recall bias should be minimal concerning their pregnancy. Recall bias is clearly likely to be greater when recalling information from the previous twelve months. Unfortunately women who required an interpreter to complete the questionnaire were excluded from the study as funding was not available. Therefore the results from the survey cannot be extrapolated to women with a non-English speaking background, although in our population these women represent less than 5% of the total population.

## Conclusion

Postnatally, most women had some knowledge of dental disease. However, this survey showed associations between lack of knowledge of dental health and dental hygiene with women with lower educational achievements and women who resided in lower socio-economic areas. Although most women appear to be informed about dental hygiene practices the majority did not attend the dentist during the previous 12 months. Whether better education on dental health in pregnancy can lead to improved dental practices, improved health and better pregnancy health outcomes requires further investigation.

## Competing interests

The author(s) declares that they have no competing interests.

## Authors' contributions

NT designed and administered the survey, under the supervision of CC. All authors interpreted the data and wrote the paper.

## Pre-publication history

The pre-publication history for this paper can be accessed here:


